# An imprinted rheumatoid arthritis methylome signature reflects pathogenic phenotype

**DOI:** 10.1186/gm444

**Published:** 2013-04-30

**Authors:** John W Whitaker, Robert Shoemaker, David L Boyle, Josh Hillman, David Anderson, Wei Wang, Gary S Firestein

**Affiliations:** 1 Department of Chemistry and Biochemistry, University of California San Diego, La Jolla, CA, USA; 2 Ignyta, Inc., San Diego, CA, USA; 3 Division of Rheumatology, Allergy and Immunology, UCSD School of Medicine, La Jolla, CA, USA

## Abstract

**Background:**

A DNA methylation signature has been characterized that distinguishes rheumatoid arthritis (RA) fibroblast like synoviocytes (FLS) from osteoarthritis (OA) FLS. The presence of epigenetic changes in long-term cultured cells suggest that rheumatoid FLS imprinting might contribute to pathogenic behavior. To understand how differentially methylated genes (DMGs) might participate in the pathogenesis of RA, we evaluated the stability of the RA signature and whether DMGs are enriched in specific pathways and ontology categories.

**Methods:**

To assess the RA methylation signatures the Illumina HumanMethylation450 chip was used to compare methylation levels in RA, OA, and normal (NL) FLS at passage 3, 5, and 7. Then methylation frequencies at CpGs within the signature were compared between passages. To assess the enrichment of DMGs in specific pathways, DMGs were identified as genes that possess significantly differential methylated loci within their promoter regions. These sets of DMGs were then compared to pathway and ontology databases to establish enrichment in specific categories.

**Results:**

Initial studies compared passage 3, 5, and 7 FLS from RA, OA, and NL. The patterns of differential methylation of each individual FLS line were very similar regardless of passage number. Using the most robust analysis, 20 out of 272 KEGG pathways and 43 out of 34,400 GO pathways were significantly altered for RA compared with OA and NL FLS. Most interestingly, we found that the KEGG 'Rheumatoid Arthritis' pathway was consistently the most significantly enriched with differentially methylated loci. Additional pathways involved with innate immunity (Complement and Coagulation, Toll-like Receptors, NOD-like Receptors, and Cytosolic DNA-sensing), cell adhesion (Focal Adhesion, Cell Adhesion Molecule), and cytokines (Cytokine-cytokine Receptor). Taken together, KEGG and GO pathway analysis demonstrates non-random epigenetic imprinting of RA FLS.

**Conclusions:**

The DNA methylation patterns include anomalies in key genes implicated in the pathogenesis of RA and are stable for multiple cell passages. Persistent epigenetic alterations could contribute to the aggressive phenotype of RA synoviocytes and identify potential therapeutic targets that could modulate the pathogenic behavior.

## Background

RA is a chronic inflammatory disease marked by synovial hyperplasia and invasion into cartilage and bone. This process is mediated, in part, by cytokines like IL-1, IL-6, and TNF that activate a broad array of cell signaling mechanisms and leads to the release of destructive enzymes [1]. Fibroblast-like synoviocytes (FLS), which form the inner lining of the synovium, play an active role in joint destruction by invading intra-articular cartilage and other support structures of the joint [2]. These mesenchymal cells normally produce hyaluronic acid and other lubricants that facilitate joint movement and a low friction environment.

FLS display an aggressive phenotype in RA that persists in long-term culture [2,3]. These imprinted cells can migrate between joints and exhibit characteristics of locally invasive transformed cells [4]. The mechanism that contribute to functional alterations in RA FLS are only partially understood and include somatic mutations, alterations in cell survival and apoptosis genes, and persistent activation of signaling pathways [3]. Epigenetic changes, including aberrant miRNA expression [5], can also contribute to the aggressive RA FLS phenotype.

More recently, a characteristic DNA methylation signature that could affect cell function and distinguishes RA from osteoarthritis (OA) FLS was discovered [6]. Differentially methylated loci (DML) involve many key genes implicated in inflammation, immune responses, cell-cell interactions, and matrix regulation. The original study defining the RA methylation pattern was performed on a relatively limited number of cell lines and did not evaluate the stability of the signature over multiple passages. For the present analysis, we increased the number of OA and RA cell lines and included normal (NL) synoviocytes. The greater number of cells permitted a focused evaluation of promoter sequences and a more detailed pathway analysis. The results demonstrate a pattern of differentially methylated pathways in RA FLS that define pathogenic processes that could permit identification of novel therapeutic targets.

## Methods

### FLS and patient phenotype

FLS were isolated from synovial tissues obtained from 11 RA and 11 OA patients at the time of joint replacement as described previously [7]. The diagnosis of RA conformed to the American College of Rheumatology 1987 revised criteria [8]. The protocol was approved by the UCSD Human Subjects Research Protection Program. Synoviocytes were used from passage 3 through 7, when FLS were a homogeneous population with < 1% CD11b, < 1% phagocytic, and < 1% FcR II positive cells. Normal human synoviocytes were provided by the San Diego Tissue Bank from autopsy specimens. Preparation of the genomic DNA from early, middle, and late passage cells (passages 3, 5, and 7, respectively) for the Infinium HumanMethylation450 BeadChip (Illumina; San Diego, CA) and calculation of methylation frequencies (Beta values) was performed as previously reported [6]. The BeadChip data from the original 11 samples in reference 6 are available through the Gene Expression Omnibus (GEO) under the accession [GSE46364].

### BeadChip processing and validation

BeadChip data were processed in R 2.15 using the methylumi and minfi packages. Data were normalized via the minfi package (preprocessIllumina function). CpGs with detection *P *values > 0.001 were filtered out. For chip validation, biologic replicates within chips were performed and demonstrated a mean correlation coefficient r^2 ^= 0.9338. Replicates between chips demonstrated a mean correlation coefficient r^2 ^= 0.9858. Regarding potential performance differences between Infinium I and II probes on the BeadChip, we reasoned that since CpGs were tested independently, probe type-specific bias would be equally present in both phenotype populations. Therefore, the risk of false-positive detection due to probe-type differences is low.

### Methylation heat maps and histograms

Methylation frequencies of previously identified RA-OA differentially methylated CpGs [6] across FLS samples were multiplied by 100 to obtain values interpreted as methylation percentages. The Euclidian distances between FLS samples and CpGs were calculated and hierarchically clustered using complete linkage. The results were visualized in a heat map using the heatmap.2 function in the gplots R package as previously described [6]. Missing values were represented with a white color. For each FLS cell line, beta value differences of the previously identified 1,859 CpG signature between passages were calculated and plotted as histograms with bins for every 0.01 interval. The bar areas were normalized so that their total sum equaled unity.

### Correlations between FLS passage number

The FLS lines were experimentally processed the same day across three BeadChips. This approach minimized intra-dataset (that is, KEGG and passage FLS datasets) batch effects like BeadChip lot, reagent lot, lab processing, or temporal variability). The tight hierarchical clustering of P5 FLS samples across the KEGG and passage datasets demonstrates that inter-dataset batch effects are minor relative to the RA methylation signature (see Results). Furthermore, validation studies of duplicate samples on the same BeadChip, on different BeadChips, or performed on different runs showed very strong correlations (data not shown). For each cell line, the Spearman Rank correlation of beta values of the previously identified 1,859 CpG signatures was calculated across passages. For each pairwise correlation calculation, CpGs with missing data were omitted. For the correlation between replicates of passage 5 comparisons were made within the same cell line. To estimate passage correlation, the Spearman correlation coefficients between passage 5 and the other passages were calculated per cell line. An average was calculated based on these correlation coefficients.

### Identifying differentially methylated genes (DMGs)

A Welch's *t*-test was used to calculate the significance of differential methylation. We chose to apply Welch's *t*-test as the samples may have unequal variance. All loci that are present on the array were tested and the resulting *P *values were converted into *q *values [9]. Additionally, the average difference in mean values at each position was calculated. Loci with *q *values < 0.05 and average mean differences > 0.1, or < -0.1, were labelled as differential methylated loci (DML). Missing values and values with detection *P *values > 0.01 were not included in this analysis. At a specific locus, if values were absent but at least three values present, for each of the two groups being considered, then comparison was made between only the existing values. If there were less than three values present in a single group, at a specific locus, then this locus was ignored from the analysis. The DMLs within the promoters of genes were identified and the DMLs were linked to the corresponding genes. The locations of refseq genes were obtained from the University of California, Santa Cruz. If a promoter contained one or more DMLs within a 3 kb window around a gene's transcription start site (TSS) (-2500 bps to +500 bps from the TSS) then the gene was labelled as a DMG.

The significance of DMG in 271 KEGG human pathways was assessed [10]. Enrichment factors (EF) were calculated as:

$$\text{EF }=\;\left(\text{total DMGs in pathway}/)\text{total genes in pathway}\right)/)\left(\text{total DMGs}/)\text{total genes in KEGG}\right)\;$$

The 'total genes in the pathway' and 'total genes in KEGG' only considered genes that are present in a KEGG pathway and are covered by the BeadChip.

The hypergeometric distribution was used to calculate enrichment *P *values that were converted into *q *values. Resulting *q *values represented the fraction of randomly selected background gene sets that were at least as enriched in genes found in the tested pathway as the DMG set. A *q *value threshold of 0.05 determined significance. The significance of 34,449 GO categories was tested in the same way. The background for the GO analysis included all the genes whose promoters are covered by the methylation array and that are present in the GO database.

## Results

### Stability of the methylome signature in RA FLS

We previously identified a methylome signature in RA comprised of 1,859 DML and predicts the phenotype of passage 5 FLS (RA *vs*. OA). One critical question to answer before performing additional pathway analysis with more cell lines is whether this signature is stable. Therefore, we assayed the methylomes of nine FLS cell lines (three RA, three OA, and three NL) at the 3rd, 5th, and 7th passage (P3, P5, and P7, respectively). Passage 3 cells were the earliest samples evaluated because passage 1 and 2 lines can include other cell types. By the 3rd passage, FLS are a homogeneous population of cells (< 1% CD11b positive, < 1% phagocytic, and < 1% FcR II and FcR III receptor positive) [2].

**Figure 1:**
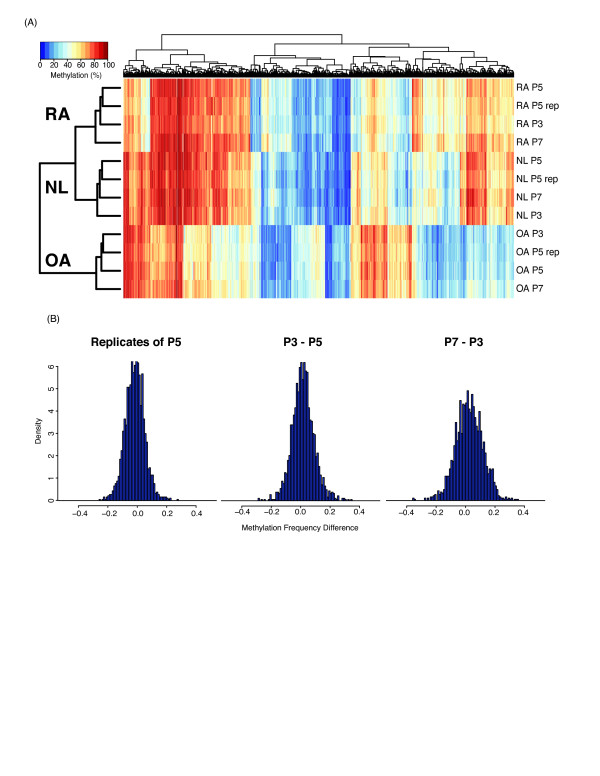
**The RA methylome signature is stable over passages**. **(A) **Hierarchical clustering RA, OA, and NL samples at P3, P5, and P7. Each column shows the methylation level at one of the 1,859 DML. Additionally, samples from P5 of Nakano *et al*. 2012 are shown as reference and are labeled as rep. **(B) **The distribution of the difference in beta values between passages for RA sample.

We first performed hierarchical clustering of RA, OA, and NL samples from the three passages. In addition, we included P5 samples from our previous study as an internal biological sample control. The RA, OA, and NL groups clustered separately at all passages, confirming that the RA methylome signature is stable over many passages (see Figure 1A for representative examples). Of note, the replicates of P5 clustered very closely and were also only slightly different from the other passages.

To further explore the stability of the RA methylome signature over time, we considered the difference in methylation percentages between different passages and the replicates of P5. Figure 1B shows that variation between replicates of P5 is comparable to the variation between P5-P3 and P5-P7. The correlation across the nine cell lines between P5 replicates is 0.950 while the correlation between P3-P5 is 0.943. These data show that P3 and P5 are as highly correlated as replicates of the same passage and that there is very little change in the methylation frequencies between P3 and P5. There is a slight decrease in correlation between P3 to P7 (0.883) as the cells approach senescence.

### Determination of differentially methylated genes in RA FLS

Having confirmed that the RA methylome signature is stable we then investigated its relationship with clinical phenotype on a systems level. Previously, we had carried out KEGG and GO analysis on a limited set of samples (six RA and five OA FLS lines). We have advanced on this previous analysis by increasing the sample size to 11 RA and 11 OA and adding six NL lines. The increased number of samples from 11 to 28 allowed us to focus on the subset of DMLs within the gene promoters (-2500 to +500 bps from the TSS). DMLs were identified by calculating *P *values using Welch's t test. Then the resulting *P *values were corrected for multiple testing to produce *q *values. Genes that contain DMLs within their promoters were labeled DMGs. An average mean difference of 0.1 or greater was required for a DML to be considered significant.

Identification of DMGs was carried out by comparing the RA samples to five combinations of OA and NL: 'RA *vs*. OA', 'RA *vs*. NL', 'RA *vs*. OA+NL' (which combines the OA and NL databases), 'RA *vs*. OA or NL' (which includes DMGs that are significant in either RA *vs*. OA or RA *vs*. NL) and 'RA *vs*. (OA or NL) or (OA+NL)' (denoted as 'combined'). We focused on comparisons that take account of both OA and NL as these are of greatest relevance to differentiating RA phenotype from non-RA. Furthermore, smaller NL sample size identified a reduced set of DMGs making this set of DMGs unsuitable for systems level analysis.

A summary of the number of genes identified in each of the comparisons is shown in Table 1 (for a full list of genes, see Supplemental Table 1). The majority (90%) of the 2,375 total genes identified (the combined) as DMGs were identified solely by comparison of RA and OA.

**Table 1:** A summary of the number of DMGs, pathways, and GO terms identified as significant

	Combined	OA+NL	OA or NL
DMGs	2,375	472	2,346

KEGG	20	19	25

GO	43	546	40

The sets were defined through the following comparisons: 'combined' combines the sets from the other two comparisons; 'OA+NL' compares RA with a combined RA and NL database; 'OA or NL' includes DMGs that are significant in either 'RA *vs*. OA' or 'RA *vs*. NL'. A complete list of DMGs is provided in Additional file 1. DMGs: differentially methylated genes.

### Determination of differentially methylated KEGG pathways in RA FLS

Having established sets of DMGs, we identified the biological pathways and gene ontologies that were enriched within the sets. To identify enriched pathways, the DMGs where mapped to the KEGG pathways and GO databases. Enrichment *P *values were then calculated using the hypergeometric distribution. Then these *P *values were corrected for multiple testing to produce *q *values. A *q *values cut-off of < 0.05 was considered significant.

A summary of the total number of significant KEGG pathways and GO categories that were found significant is given in Table 2. The 221 additional DMGs that are included in the Combined set have a significant effect on KEGG pathway enrichment and result in 13 additional pathways identified as significantly enriched compared with the previous published analysis on a limited dataset.

**Table 2:** KEGG pathways that enriched with genes that contain DML within their promoters

**Comparison name**	**Combined**		**OA+NL**		**OA or NL**	
**Comparison description**	**RA vs (OA or NL) or (OA+NL)**		**RA vs OA+NL**		**RA vs OA or NL**	
**Pathway name**	**EF (*q*-value)**	**# of DMGs (%)**	**EF (*q*-value)**	**# of DMGs (%)**	**EF (*q*-value)**	**# of DMGs (%)**
			
Rheumatoid arthritis	2.5 (0.00266)	24 (27.0)	6.5 (1.91e-05)	12 (13.5)	2.3 (0.00467)	22 (24.7)
NOD-like receptor signaling pathway	2.5 (0.00741)	16 (27.6)	5.8 (0.0014)	7 (12.1)	2.3 (0.0374)	14 (24.1)
Cell adhesion molecules (CAMs)	1.8 (0.0294)	25 (19.4)	3.0 (0.0232)	8 (6.2)	1.8 (0.0343)	25 (19.4)
Focal adhesion	1.6 (0.0287)	35 (17.6)			1.6 (0.0321)	35 (17.6)
Cytosolic DNA-sensing pathway			4.6 (0.0202)	5 (9.6)		
Cytokine-cytokine receptor interaction			2.3 (0.0231)	12 (4.8)		
Toll-like receptor signaling pathway	1.9 (0.0377)	19 (20.7)				
Complement and coagulation cascades	2.0 (0.0494)	15 (21.7)				
Staphylococcus aureus infection	2.9 (0.00379)	16 (31.4)	4.7 (0.019)	5 (9.8)	2.9 (0.00457)	16 (31.4)
Graft-versus-host disease	2.7 (0.0211)	11 (29.7)	10.4 (2.66e-05)	8 (21.6)	2.5 (0.0468)	10 (27.0)
Allograft rejection	2.6 (0.031)	10 (28.6)	9.6 (0.000159)	7 (20.0)	2.7 (0.0389)	10 (28.6)
Leishmaniasis	2.0 (0.0451)	15 (22.1)	4.9 (0.00304)	7 (10.3)	2.1 (0.048)	15 (22.1)
Toxoplasmosis	1.7 (0.0435)	24 (18.8)	3.4 (0.00759)	9 (7.0)	1.7 (0.046)	24 (18.8)
Viral myocarditis	2.6 (0.00392)	19 (27.9)			2.6 (0.00457)	19 (27.9)
Dilated cardiomyopathy	2.3 (0.00489)	22 (24.7)			2.3 (0.00467)	22 (24.7)
Hypertrophic cardiomyopathy (HCM)	2.3 (0.00487)	21 (25.3)			2.2 (0.00904)	20 (24.1)
Aldosterone-regulated sodium reabsorption	2.8 (0.00761)	13 (31.0)			2.9 (0.00814)	13 (31.0)
Influenza A	1.7 (0.0308)	29 (18.4)	4.0 (0.000283)	13 (8.2)		
Type II diabetes mellitus	2.5 (0.0185)	13 (27.7)			2.6 (0.0203)	13 (27.7)
Carbohydrate digestion and absorption	2.6 (0.0225)	12 (27.9)			2.4 (0.0474)	11 (25.6)
Type I diabetes mellitus			8.2 (0.000269)	7 (17.1)		
Asthma			10.3 (0.000274)	6 (21.4)		
Tuberculosis			3.8 (0.000386)	13 (7.9)		
Antigen processing and presentation			5.7 (0.000668)	8 (11.9)		
Phagosome			3.7 (0.00131)	11 (7.7)		
Autoimmune thyroid disease			6.9 (0.00152)	6 (14.3)		
Intestinal immune network for IgA production			6.3 (0.00233)	6 (13.0)		
Chagas disease (American trypanosomiasis)	2.0 (0.0206)	22 (21.4)				
Prion diseases			5.5 (0.0246)	4 (11.4)		
African trypanosomiasis	2.6 (0.031)	10 (28.6)				

KEGG pathways that are found as significantly enriched (*q *< 0.05) in three comparisons are shown. Eight pathways especially relevant to RA are placed in the top part of the table. Within each section, the number of significant comparisons orders the pathways. If multiple pathways have the same number of comparison then an average *q *values is used for ordering. # denotes the number of DMGs in the selected pathway.

**Figure 2:**
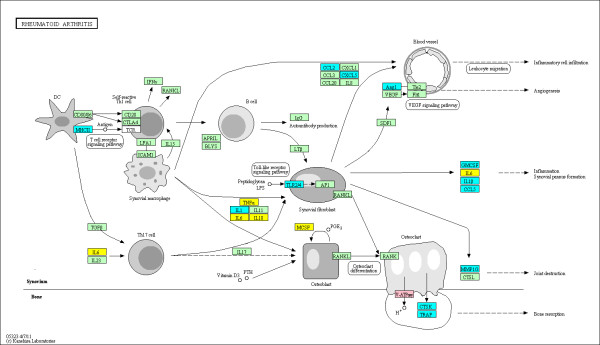
**DMGs in the KEGG RA pathway**. The methylation status at the promoters of genes within the pathway is shown. The coloring scheme is as follows: yellow are hyper-methylated in RA, blue are hypo-methylated in RA, pink contain both hyper and hypo loci in their promoters, and green contain no significantly DML in their promoters.

**Figure 3:**
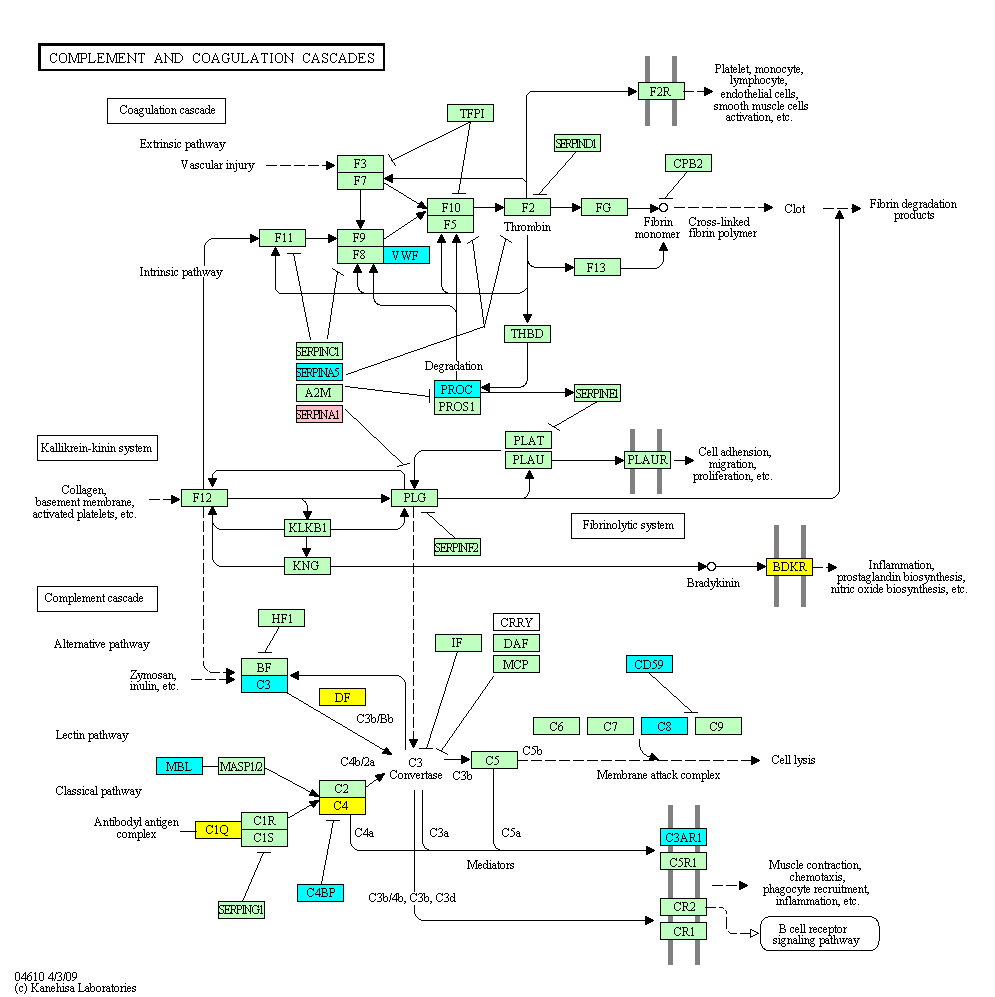
**DMGs in the KEGG 'Complement and coagulation cascades' pathway**. The methylation status at the promoters of genes within the pathway is shown. The coloring scheme is as follows: yellow are hyper-methylated in RA, blue are hypo-methylated in RA, pink contain both hyper and hypo loci in their promoters, and green contain no significantly DML in their promoters.

**Figure 4:**
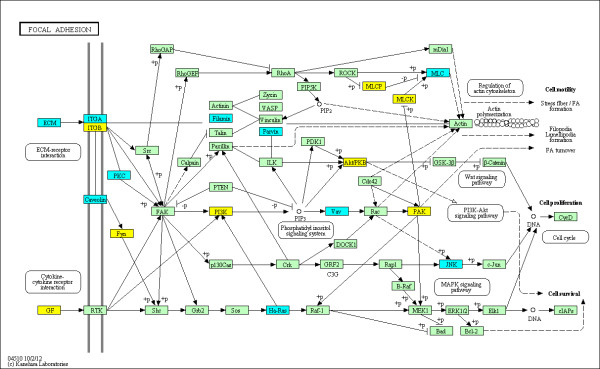
**DMGs in the KEGG 'Focal Adhesion' pathway**. The methylation status at the promoters of genes within the pathway is shown. The coloring scheme is as follows: yellow are hyper-methylated in RA, blue are hypo-methylated in RA, and green contain no significantly DML in their promoters. Note that the following extracellular matrix (ECM) genes not specified in the figure are hypo-methylated: COL1A1, COL1A2, COL2A1, COMP, LAMA2, LAMB3, and VWF.

**Figure 5:**
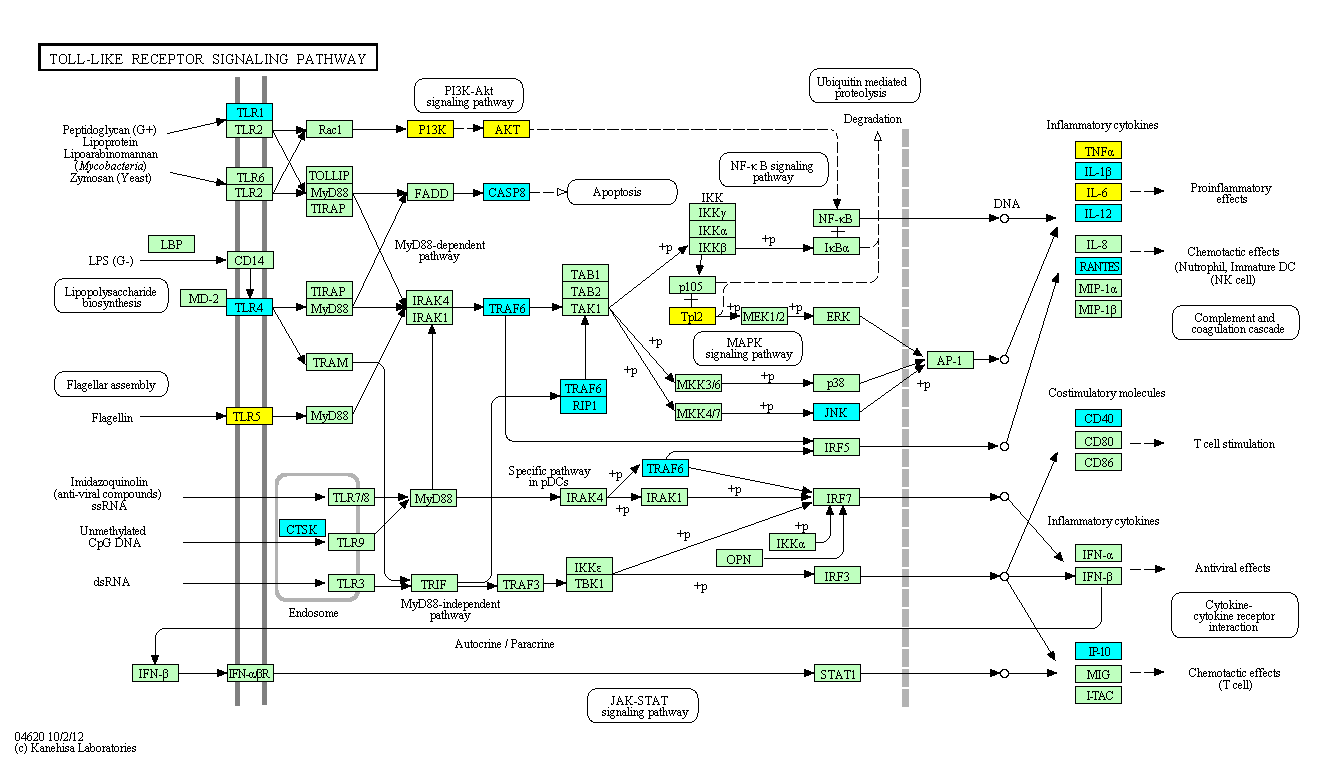
**DMGs in the KEGG 'Toll-like receptor signaling' pathway**. The methylation status at the promoters of genes within the pathway is shown. The coloring scheme is as follows: yellow are hyper-methylated in RA, blue are hypo-methylated in RA and green contain no significantly DML in their promoters.

The enriched KEGG pathways out of a total of 271 pathways evaluated are summarized in Table 3 using the various groupings described above. The top ranked pathway is the KEGG 'rheumatoid arthritis' pathway (see Figure 2) with 2.47-fold enrichment (*P *= 1.729e-05 and *q *= 0.0027) and 24 out of 89 genes identified as DMGs in the union set. This confirms that the observed alterations in DNA methylation are highly relevant to RA. Furthermore, eight additional immunological pathways relevant to RA were also identified as significantly enriched with DMGs in RA FLS. For example, KEGG 'Complement and coagulation cascades' pathway (see Figure 3) is 1.99-fold enriched (*P *= 0.0064 and *q *= 0.0494) with 15 out of 69 genes labeled as DMGs in the union set. The KEGG 'Focal Adhesion' pathway (see Figure 4) is 1.61-fold enriched (*P *= 0.0027 and *q *= 0.0420) with 35 out of 199 genes labeled as DMGs in the union set. The KEGG 'Toll-like receptor signaling' pathway (see Figure 5) is 1.89-fold enriched (*P *= 0.0042 and *q *= 0.0377) with 19 out of 92 genes labeled as DMGs in the union set.

**Table 3:** Gene ontology terms that are enriched in two or more sets of genes

**Comparison name**		**Combined**		**OA+NL**		**OA or NL**	
**Comparison description**		**RA vs OA or NL or OA+NL**		**RA vs OA+NL**		**RA vs OA or NL**	
**Pathway name**	**GO type**	**EF (*q*-value)**	**# of DMGs (%)**	**EF (*q*-value)**	**# of DMGs (%)**	**EF (*q*-value)**	**# of DMGs (%)**
				
positive regulation of ERK1 and ERK2 cascade	BP	3.5 (0.000494)	18 (38.3)	9.0 (0.000111)	9 (19.1)	3.3 (0.00106)	17 (36.2)
proteinaceous extracellular matrix	CC	2.2 (7.07e-05)	48 (24.2)	3.3 (0.00287)	14 (7.1)	2.1 (0.000254)	46 (23.2)
signal transduction	BP	1.4 (0.000717)	174 (15.4)	1.5 (0.0263)	37 (3.3)	1.4 (0.000713)	172 (15.2)
extracellular matrix	CC	2.3 (0.00282)	30 (25.0)	2.3 (0.0441)	6 (5.0)	2.3 (0.00192)	30 (25.0)
inflammatory response	BP	1.8 (0.00821)	45 (20.1)	2.3 (0.0301)	11 (4.9)	1.8 (0.0106)	44 (19.6)
fibroblast growth factor receptor signaling'	BP	9.1 (0.02)	4 (100.0)	23.5 (0.0176)	2 (50.0)	9.2 (0.0177)	4 (100.0)
MHC class II protein complex	CC	4.5 (0.0346)	7 (50.0)	20.1 (9.47e-05)	6 (42.9)	4.6 (0.0303)	7 (50.0)
focal adhesion	CC	2.1 (0.0176)	26 (23.6)	2.6 (0.0418)	6 (5.5)	2.2 (0.0136)	26 (23.6)
Rho guanyl-nucleotide exchange factor activity	MF	2.3 (0.0443)	18 (25.4)	3.3 (0.0375)	5 (7.0)	2.3 (0.0377)	18 (25.4)
regulation of Rho protein signal transduction	BP	2.3 (0.0481)	18 (25.0)	3.3 (0.038)	5 (6.9)	2.3 (0.0412)	18 (25.0)
positive regulation of inflammatory response	BP	3.3 (0.0173)	12 (36.4)			3.3 (0.0141)	12 (36.4)
cell adhesion	BP	1.4 (0.0425)	84 (15.7)			1.4 (0.0404)	83 (15.5)
extracellular region	CC	1.6 (2.4e-13)	306 (17.2)	1.8 (0.000356)	67 (3.8)	1.6 (4.11e-13)	302 (16.9)
extracellular space	CC	1.6 (2.91e-06)	133 (18.1)	2.2 (0.000971)	34 (4.6)	1.6 (7.7e-06)	130 (17.7)
immune response	BP	1.9 (0.000153)	64 (21.0)	3.1 (0.000722)	20 (6.6)	1.9 (0.000352)	62 (20.3)
plasma membrane	CC	1.3 (4.6e-08)	467 (14.3)	1.4 (0.00269)	99 (3.0)	1.3 (6.35e-08)	461 (14.1)
Z disc	CC	3.5 (0.00133)	16 (38.1)	5.6 (0.0161)	5 (11.9)	3.5 (0.00099)	16 (38.1)
scavenger receptor activity	MF	3.5 (0.00104)	16 (39.0)	4.6 (0.0327)	4 (9.8)	3.6 (0.000814)	16 (39.0)
odontogenesis	BP	4.2 (0.00175)	12 (46.2)	5.4 (0.0374)	3 (11.5)	4.2 (0.00132)	12 (46.2)
integral to plasma membrane	CC	1.4 (0.00501)	149 (15.2)	1.6 (0.0271)	33 (3.4)	1.4 (0.0102)	145 (14.8)
multicellular organismal development	BP	1.5 (0.000606)	143 (16.1)	1.4 (0.0435)	27 (3.0)	1.5 (0.000332)	143 (16.1)
structural molecule activity	MF	1.9 (0.00594)	42 (20.9)	2.1 (0.0414)	9 (4.5)	1.9 (0.0041)	42 (20.9)
positive regulation of cell proliferation	BP	1.6 (0.019)	58 (17.8)	2.9 (0.00105)	20 (6.2)	1.6 (0.0315)	56 (17.2)
positive regulation of interferon-gamma production	BP	3.2 (0.0439)	10 (35.7)	10.1 (0.00104)	6 (21.4)	3.3 (0.0383)	10 (35.7)
skeletal muscle contraction	BP	5.5 (0.0292)	6 (60.0)	9.4 (0.0378)	2 (20.0)	5.5 (0.0256)	6 (60.0)
sarcolemma	CC	2.5 (0.0352)	17 (27.0)	3.7 (0.0326)	5 (7.9)	2.5 (0.0296)	17 (27.0)
structural constituent of muscle	MF	2.7 (0.0487)	13 (29.5)	4.3 (0.0353)	4 (9.1)	2.7 (0.0423)	13 (29.5)
actin binding	MF	1.6 (0.0497)	49 (17.5)	2.0 (0.0373)	12 (4.3)	1.6 (0.0404)	49 (17.5)
myeloid cell differentiation	BP	4.2 (0.0456)	7 (46.7)	6.3 (0.0434)	2 (13.3)	4.3 (0.0403)	7 (46.7)
sarcomere	CC	3.3 (0.00145)	17 (36.2)			3.3 (0.00106)	17 (36.2)
skin morphogenesis	BP	9.1 (0.0036)	5 (100.0)			9.2 (0.00292)	5 (100.0)
potassium channel regulator activity	MF	3.8 (0.0172)	10 (41.7)			3.8 (0.0141)	10 (41.7)
response to organic cyclic compound	BP	2.2 (0.0177)	25 (24.0)	3.2 (0.0274)	7 (6.7)		
response to mechanical stimulus	BP	2.8 (0.0383)	13 (31.0)	5.6 (0.0161)	5 (11.9)		
response to glucocorticoid stimulus	BP	2.3 (0.0306)	20 (25.3)	3.6 (0.0273)	6 (7.6)		
response to prostaglandin E stimulus	BP	6.5 (0.0313)	5 (71.4)			6.6 (0.0277)	5 (71.4)
regulation of muscle contraction	BP	5.0 (0.0433)	6 (54.5)			5.0 (0.0383)	6 (54.5)
epidermis development	BP	2.2 (0.0448)	19 (24.7)	3.0 (0.0402)	5 (6.5)		
ion transport	BP	1.4 (0.0437)	84 (15.7)			1.4 (0.0415)	83 (15.5)
PR of GMSFP'	BP	7.3 (0.0493)	4 (80.0)			7.4 (0.0441)	4 (80.0)
troponin complex	CC	5.7 (0.0495)	5 (62.5)			5.7 (0.0442)	5 (62.5)
phosphatase binding	MF	5.7 (0.0495)	5 (62.5)			5.7 (0.0442)	5 (62.5)

GO categories that are significantly enriched (*q *< 0.05) during three comparisons are shown. Twelve pathways especially relevant to RA are placed in the top part of the table while other pathways are show at the bottom. Within each section, the number of significant comparisons orders the pathways. If multiple GO categories are significantly enriched in the same number of comparison then an average *q *values is used for ordering. Only GO categories found as significantly enriched in three or more comparisons are shown. # denotes the number of DMGs in the selected pathway. The GO category 'positive regulation of granulocyte macrophage colony-stimulating factor production' is abbreviated to 'PR of GMSFP' and category 'regulation of fibroblast growth factor receptor signaling pathway' is abbreviated to 'fibroblast growth factor receptor signaling'. The GO types stand for: BP: biological process; CC: cellular component; MF: molecular function.

Certain KEGG pathways and GO categories are significantly enriched in 'OA+NL' or 'OA or NL' but are not significantly enriched in 'combined'. This can occur because the increase in the total number of significant genes can affect the enrichment of a subset of genes within a particular pathway. For example, the KEGG pathway 'cytosolic DNA sensing' is significantly enriched in 'OA+NL' but not in combined despite the 'OA+NL' containing five DMGs in the pathway whereas 'combined' contains eight. This occurs because the total number of DMGs increases from 472 in 'OA+NL' to 2,375 in 'combined'.

### Determination of differentially methylated GO pathways in RA FLS

The enriched GO categories out of the full 34,449 category dataset are summarized in Table 4 where 10 categories especially relevant to RA are listed at the top of the table. Of particular interest is the overlap with KEGG because of the preponderance of matrix, adhesion, and signaling GO categories.

## Discussion

Fibroblast-like synoviocytes (FLS), which form the synovial intimal lining, play an integral role in the pathogenesis of RA by producing cytokines, small molecule mediators, and proteases [1]. The FLS are responsible for cartilage damage by virtue of their ability to adhere to and invade the cartilage extracellular matrix [11]. Rheumatoid FLS exhibit a unique aggressive phenotype that contributes to the cytokine milieu and joint destruction [12]. Functional studies suggest that RA cells are imprinted *in situ *and maintain these features after many passages in tissue culture. For example, RA FLS, unlike OA or NL synoviocytes, invade cartilage explants in SCID mice [13]. RA FLS can grow under anchorage-independent conditions, are less susceptible to contact inhibition, and resistant to apoptosis [14,15].

DNA methylation could play a critical role in joint damage by epigenetic imprinting FLS in RA. Evidence of abnormal methylation and a distinct methylation signature was recently described in RA synoviocytes [6]. Using a limited number of cell lines, hyper- and hypomethylated CpG sites were demonstrated in nearly 2,000 loci located in 1,200 genes. Methylation status determined by array analysis was confirmed by bisulfite sequencing and correlated with gene expression in that study. Additional analysis identified over 200 genes with multiple differentially methylated loci. Preliminary KEGG and GO analysis suggested that pathways involved with inflammation, matrix regulation, and immune responses are differentially methylated. The present study greatly expands upon the initial dataset by doubling the number of RA and OA FLS and adding normal FLS. The increased sample size also allowed us to focus our analysis on differentially methylated promoter sites. These data refined the KEGG and GO analysis and led to identification of additional key pathways implicated in disease.

Before extending our computational studies, we determined if the RA methylation signature is stable. Comparison of the methylation patterns between different passages of RA, OA, and NL FLS showed that the signature changes very little over time. These results correlate with previous studies demonstrating that the FLS transcriptome is also stable in culture [2,16]. By the 7th passage, a slight increase in methylation variability was detected, but the correlation was only slightly lower than between replicates of the same passage. Furthermore, the most significant methylation changes that may be associated with pathogenic processes appear to be very stable over time in FLS lines. These data suggest that the majority of the variation is a result of noise in the bead array assay.

Methylome stability has been observed with other long-term cultured cell lines. For instance, multiple passages of the cell lines IMR90 (human fetal lung fibroblast) and H1 (human embryonic stem cells) showed high concordance [17]. The methylome is not immutable, however, and can be modulated by the environment, different developmental stages [18], or prolonged tissue culture [19]. For example, dynamic changes in methylome occur in human embryonic stem cells, a fibroblastic differentiated derivative of the human embryonic stem cells, and neonatal fibroblasts. Immortalized fibroblasts that evolve separately over 300 generations show stochastic methylation changes. Despite the random nature of changes, they resulted in a deterministic remodeling of the methylome that correlates with histone modification and CTCF binding.

The stability of the RA methylome signature from the earliest possible passage (P3) to cells approaching senescence (P7) suggests that it is imprinted in FLS rather than a transient phenomenon. However, it does not tell us the origin of the RA methylome signature. In particular, we are still unsure whether the imprinting predates disease, is brought about by interaction with the pre-RA synovium environment, or is modified by RA in a way that influences the behavior of FLS. Our recent study show that DNMT expression and function are suppressed in FLS when exposed to IL-1 [20]. However, this effect is transient and is reversed 2 weeks after the cytokine is removed from the cultures. Therefore, cytokines can potentially contribute to altered DNA methylation in FLS but probably do not account for the long-lasting effects. Evaluation of FLS from patients at high risk for RA but without clinical disease would be needed to resolve this issue with greater certainty. Also, specificity for RA, as opposed to other forms of inflammatory arthritis, will require evaluation of FLS from other forms of synovitis.

Systems level analysis of the RA methylome signature in our expanded dataset reveals that DMLs are highly enriched in the promoters of genes that belong to characteristic functional categories. Because we had three distinct cell populations, we used several different ways to evaluate how RA differed from OA and/or NL FLS. There were some differences in the genes and pathways that were differentially methylated depending on how the phenotypes were grouped. Relatively broad agreement among the various types of analyses was observed. The most gratifying was the RA pathway in the KEGG analysis, which was found in the three most robust comparisons and confirmed the relevance of our analysis to this disease.

We also found the enrichment of many pathways that are related to the RA phenotype and immune responses. The KEGG and GO analysis of differentially methylated pathways is, therefore, non-random and likely essential for establishment or maintenance of the RA FLS phenotype. Even many of the seemingly less relevant categories that were identified as enriched result from overlap in sets of genes that are shared with pathways that are more clearly relevant to RA. For example, the pathway '*Staphylococcus aureus *infection' contains 16 DMGs; however, six of these are also present in the RA pathway.

The nature of the RA-associated pathways enriched in our KEGG and GO analysis provides clues regarding the pathogenesis of the disease. For example, the role of complement is well documented in animal studies [21]. This innate immune mechanism is also strongly implicated in the initiation and acute inflammatory reaction of RA [22]. Several complement components are produced by FLS in the intimal lining as well as cultured synoviocytes [23]. The pathway analysis indicates that regulation of complement is abnormal in addition to the fact that it is robustly consumed in the joints.

Altered methylation and, presumably, gene regulation in FLS is also observed for many other components of innate immunity. Notably, TLR, cytosolic DNA-sensing, and NOD-like receptor pathways are significantly enriched in the KEGG analysis for differentially methylated genes. Each pathway has been implicated as fundamental mechanisms that regulate the inflammatory response in RA [24-26]. Genes regulating innate immunity are emerging as potential therapeutic targets for RA, and the methylation data supports their participation in rheumatoid synovitis. Similarly, multiple cytokines are differentially methylated in the RA pathway and in the cytokine-cytokine receptor pathway, including TNF and other critical mediators of RA. Additional types of pathways involving inflammation, host defense, and immune responses are enriched in the GO analysis and are consistent with the insights gleaned from the KEGG pathways.

The data suggest that pathway analysis can provide other clues for disease pathogenesis and novel therapeutic interventions. For example, cell-cell and cell-matrix interactions are differentially methylated in the focal adhesion pathway and the cell adhesion molecule pathways. Several potential therapeutic targets are within these pathways and could be explored for diseases like RA. The specific genes that are differentially methylated are not necessarily the ones that should be the focus of drug development. Instead, more attractive proteins in the pathway that are more amenable to inhibition or modulation could be selected and achieve the same result.

## Conclusions

An expanded dataset evaluating differentially methylated pathways confirmed the limited data and greatly extended the number of pathways that are enriched in RA. The relative stability of the signature was also demonstrated, supporting the notion that the cells are imprinted rather than merely reflecting transient cytokine effects. These data can provide insights into the pathogenesis of RA as well as identifying potential therapeutic targets.

## Abbreviations

DMG: differentially methylated gene; DML: differentially methylated loci; EF: enrichment factor; FLS: fibroblast like synoviocytes; GO: gene ontology; KEGG: Kyoto Encyclopedia of Genes and Genomes; NL: normal; OA: osteoarthritis; P3: passage 3; P5: passage 5; P7: passage 7; RA: rheumatoid arthritis.

## Competing interests

Dr. Firestein and Dr. Wang serve on the Scientific Advisory Board of Ignyta, Inc. and have equity positions. Dr. Anderson and Dr. Shoemaker are employees of Ignyta, Inc. The remaining authors declare that they have no competing interests.

## Authors' contributions

Conception and design: JWW, RS, DLB, DA, WW, and GSF. Acquisition of data: JWW, RS, and JH. Analysis and interpretation of data: JWW, RS, DA, WW, and GSF. Writing manuscript: JWW, WW, and GSF. All authors read and approved the final manuscript.

## Supplementary Material

Additional File 1 **Additional data File 1 is a table listing the three sets of differentially methylated genes**. Click here for file

## References

[B1] Firestein GS Evolving concepts of rheumatoid arthritis. Nature 2003 423 356 361 10.1038/nature01661 12748655

[B2] Bartok B Firestein GS Fibroblast-like synoviocytes: key effector cells in rheumatoid arthritis. Immunol Rev 2010 233 233 255 10.1111/j.0105-2896.2009.00859.x 20193003 PMC2913689

[B3] Bottini N Firestein GS Duality of fibroblast-like synoviocytes in RA: passive responders and imprinted aggressors. Nat Rev Rheumatol 2013 9 24 33 23147896 10.1038/nrrheum.2012.190 PMC3970924

[B4] Lefevre S Knedla A Tennie C Kampmann A Wunrau C Dinser R Korb A Schnaker EM Tarner IH Robbins PD Evans CH Sturz H Steinmeyer J Gay S Scholmerich J Pap T Muller-Ladner U Neumann E Synovial fibroblasts spread rheumatoid arthritis to unaffected joints. Nat Med 2009 15 1414 1420 10.1038/nm.2050 19898488 PMC3678354

[B5] Filkova M Jungel A Gay RE Gay S MicroRNAs in rheumatoid arthritis: potential role in diagnosis and therapy. BioDrugs 2012 26 131 141 10.2165/11631480-000000000-00000 22494429

[B6] Nakano K Whitaker JW Boyle DL Wang W Firestein GS DNA methylome signature in rheumatoid arthritis. Ann Rheum Dis 2013 72 110 117 10.1136/annrheumdis-2012-201526 22736089 PMC3549371

[B7] Rosengren S Firestein GS Boyle DL Measurement of inflammatory biomarkers in synovial tissue extracts by enzyme-linked immunosorbent assay. Clin Diagn Lab Immunol 2003 10 1002 1010 14607859 10.1128/CDLI.10.6.1002-1010.2003 PMC262451

[B8] Arnett FC Edworthy SM Bloch DA McShane DJ Fries JF Cooper NS Healey LA Kaplan SR Liang MH Luthra HS Medsger TA Mitchell DM Neustadt DH Pinals RS Schaller JG Sharp JT Wilder RL Hunder GG The American Rheumatism Association 1987 revised criteria for the classification of rheumatoid arthritis. Arthritis Rheum 1988 31 315 324 10.1002/art.1780310302 3358796

[B9] Storey JD Tibshirani R Statistical significance for genomewide studies. Proc Natl Acad Sci USA 2003 100 9440 9445 10.1073/pnas.1530509100 12883005 PMC170937

[B10] Kanehisa M Goto S Sato Y Furumichi M Tanabe M KEGG for integration and interpretation of large-scale molecular data sets. Nucleic Acids Res 2012 40 D109 114 10.1093/nar/gkr988 22080510 PMC3245020

[B11] Lee DM Kiener HP Agarwal SK Noss EH Watts GF Chisaka O Takeichi M Brenner MB Cadherin-11 in synovial lining formation and pathology in arthritis. Science 2007 315 1006 1010 10.1126/science.1137306 17255475

[B12] Firestein GS Invasive fibroblast-like synoviocytes in rheumatoid arthritis. Passive responders or transformed aggressors?. Arthritis Rheum 1996 39 1781 1790 10.1002/art.1780391103 8912499

[B13] Muller-Ladner U Kriegsmann J Franklin BN Matsumoto S Geiler T Gay RE Gay S Synovial fibroblasts of patients with rheumatoid arthritis attach to and invade normal human cartilage when engrafted into SCID mice. Am J Pathol 1996 149 1607 1615 8909250 PMC1865262

[B14] Lafyatis R Remmers EF Roberts AB Yocum DE Sporn MB Wilder RL Anchorage-independent growth of synoviocytes from arthritic and normal joints. Stimulation by exogenous platelet-derived growth factor and inhibition by transforming growth factor-beta and retinoids. J Clin Invest 1989 83 1267 1276 10.1172/JCI114011 2784799 PMC303817

[B15] Baier A Meineckel I Gay S Pap T Apoptosis in rheumatoid arthritis. Curr Opin Rheumatol 2003 15 274 279 10.1097/00002281-200305000-00015 12707581

[B16] Hirth A Skapenko A Kinne RW Emmrich F Schulze-Koops H Sack U Cytokine mRNA and protein expression in primary-culture and repeated-passage synovial fibroblasts from patients with rheumatoid arthritis. Arthritis Res 2002 4 117 125 10.1186/ar391 11879547 PMC83845

[B17] Lister R Pelizzola M Dowen RH Hawkins RD Hon G Tonti-Filippini J Nery JR Lee L Ye Z Ngo QM Edsall L Antosiewicz-Bourget J Stewart R Ruotti V Millar AH Thomson JA Ren B Ecker JR Human DNA methylomes at base resolution show widespread epigenomic differences. Nature 2009 462 315 322 10.1038/nature08514 19829295 PMC2857523

[B18] Laurent L Wong E Li G Huynh T Tsirigos A Ong CT Low HM Kin Sung KW Rigoutsos I Loring J Wei CL Dynamic changes in the human methylome during differentiation. Genome Res 2010 20 320 331 10.1101/gr.101907.109 20133333 PMC2840979

[B19] Landan G Cohen NM Mukamel Z Bar A Molchadsky A Brosh R Horn-Saban S Zalcenstein DA Goldfinger N Zundelevich A Gal-Yam EN Rotter V Tanay A Epigenetic polymorphism and the stochastic formation of differentially methylated regions in normal and cancerous tissues. Nat Genet 2012 44 1207 1214 10.1038/ng.2442 23064413

[B20] Nakano K Boyle DL Firestein GS Regulation of DNA methyltransferases and DNA methylation in rheumatoid arthritis synoviocytes. J Immunol 2012 190 1297 1303 23277489 10.4049/jimmunol.1202572 PMC3552038

[B21] Kyburz D Corr M The KRN mouse model of inflammatory arthritis. Springer Semin Immunopathol 2003 25 79 90 10.1007/s00281-003-0131-5 12904893

[B22] Maciejewska Rodrigues H Jungel A Gay RE Gay S Innate immunity, epigenetics and autoimmunity in rheumatoid arthritis. Mol Immunol 2009 47 12 18 10.1016/j.molimm.2009.01.010 19232437

[B23] Firestein GS Paine MM Littman BH Gene expression (collagenase, tissue inhibitor of metalloproteinases, complement, and HLA-DR) in rheumatoid arthritis and osteoarthritis synovium. Quantitative analysis and effect of intraarticular corticosteroids. Arthritis Rheum 1991 34 1094 1105 10.1002/art.1780340905 1657009

[B24] Brentano F Kyburz D Schorr O Gay R Gay S The role of Toll-like receptor signalling in the pathogenesis of arthritis. Cell Immunol 2005 233 90 96 10.1016/j.cellimm.2005.04.018 15963480

[B25] Carrion M Juarranz Y Perez-Garcia S Jimeno R Pablos JL Gomariz RP Gutierrez-Canas I RNA sensors in human osteoarthritis and rheumatoid arthritis synovial fibroblasts: immune regulation by vasoactive intestinal peptide. Arthritis Rheum 2011 63 1626 1636 10.1002/art.30294 21337319

[B26] Ospelt C Brentano F Jungel A Rengel Y Kolling C Michel BA Gay RE Gay S Expression, regulation, and signaling of the pattern-recognition receptor nucleotide-binding oligomerization domain 2 in rheumatoid arthritis synovial fibroblasts. Arthritis Rheum 2009 60 355 363 10.1002/art.24226 19180502

